# STRUCTURAL AND FUNCTIONAL ASPECTS OF SOCIAL NETWORKS AND EMOTIONAL WELL-BEING AMONG LGBT OLDER ADULTS

**DOI:** 10.1093/geroni/igad104.1733

**Published:** 2023-12-21

**Authors:** Anyah Prasad, Jeffrey Burr, Edward Miller, Karen Fredriksen-Goldsen

**Affiliations:** University of Massachusetts Boston, Boston, Massachusetts, United States; University of Massachusetts Boston, Boston, Massachusetts, United States; University of Massachusetts Boston, Boston, Massachusetts, United States; University of Washington, Seattle, Washington, United States

## Abstract

Network size and composition are structural aspects and perceived support is a functional aspect of social networks. How these social network characteristics are related to LGBT older adults’ emotional wellbeing is not well understood. We investigated these relationships using data from The Caring and Aging with Pride study, a cross-sectional survey of 2,560 LGBT Americans aged 50 years and above, employing a series of mediated and moderated-mediated regression analyses. Results indicated that larger social networks were associated with more perceived support and perceived support partially mediated the association between network size and emotional wellbeing. Results also showed that stress was associated with poor emotional wellbeing via depletion of perceived support but less so when LGBT older adults were embedded in larger networks. These findings are in line with the Convoy Model of Social Relationships and The Stress Process Model; further, the results support Kondrat and colleagues’ suggestion that social network size has a moderating role, while social support has a mediating role between stress and health. Also, the Theory of Homophily postulates that shared identity may enhance group cohesion and feeling supported. Accordingly, we observed that perceived support and its mediation role were stronger with LGBT and older network members compared to non-LGBT and younger network members. A larger network of non-LGBT older adults had a more direct beneficial association with LGBT older adults’ emotional wellbeing. Our observations have practical implications for programs that aim to support LGBT older adults’ emotional wellbeing by strengthening social support through their social networks.

